# Biting patterns and seasonality of anopheles gambiae sensu lato and anopheles funestus mosquitoes in Kamuli District, Uganda

**DOI:** 10.1186/1756-3305-6-340

**Published:** 2013-12-05

**Authors:** Fredrick G Kabbale, Anne M Akol, John B Kaddu, Ambrose W Onapa

**Affiliations:** 1 Department of Biological Sciences, College of Natural Sciences, Makerere University, P.O. Box 7062, Kampala, Uganda; 2 Envision/NTD Program, RTI, Kampala, Uganda

**Keywords:** Long Lasting Insecticide-treated bed nets (LLINs), Malaria, Anthropophilic mosquitoes, Culicidae

## Abstract

**Background:**

We investigated the biting patterns and seasonal abundances of *Anopheles gambiae s.l*. and *An. funestus* mosquitoes in Kamuli District, Uganda.

**Methods:**

Hourly indoor and outdoor catches of human biting mosquitoes were sampled from 19.00 to 07.00 hours for four consecutive nights each month using bed net traps in forty-eight houses randomly selected from Bugabula county where insecticide-treated bed nets (ITNs) had been used for at least five years and Budiope county where ITNs had not been used. The indoor and outdoor human-biting fractions, time of biting of the anophelines and climatic data were recorded from January to December 2010. Data were analysed using Multi-way analysis of variance, Kruskal-wallis rank sum test and Pearson correlation. The number of mosquitoes caught biting humans and resting indoors, the indoor and outdoor human biting densities and biting rates during different hours of the night, and mosquito abundances for a twelve-month sampling period in both zones are reported.

**Results:**

Approximately four times more *Anopheles* mosquitoes were caught biting humans in Budiope County than in the Bugabula zone, with *An. gambiae s. l*. catches exceeding those of *An. funestus*. In both zones, peak night biting occurred between 23.00 and 05.00 hours. The majority of bites occurred between 03.00 and 06.00 hours for both *Anopheles gambiae s. l*. and *funestus* group. Outdoor biting densities of *Anopheles gambiae s. l*. exceeded the indoor biting densities throughout the night in both zones, while the indoor and outdoor human biting densities of *An. funestus* group were apparently equal. The outdoor and indoor human biting rates were similar in both zones. In Bugabula county, the abundance of *An. gambiae s.l*. was rainfall-dependent, while the *An. funestus* group could thrive with or without rain fall. In Budiope county, both *An. gambiae s.l*. and *An. funestus* mosquitoes thrived all year round regardless of the amount of rainfall.

**Conclusion:**

Considering the biting patterns, and seasonal abundances exhibited by *Anopheles gambiae s.l*. and *An. funestus* mosquitoes in Kamuli district, intensive use of ITNs combined with indoor residual spraying, environmental management and improved house designs in the context of integrated vector management may be the appropriate vector control strategy.

## Background

Malaria remains the leading cause of morbidity and mortality in Uganda where 95% of the country is malaria endemic [1]. Malaria accounts for up to 45% of all outpatient visits, 25% of all admissions at hospitals and 9 to 14% of all hospital deaths [2]. According to a 2012 World Malaria report, Uganda is one of the highest malaria burden countries in the African region with an incidence rate of more than one case per 1000 population per year in the high transmission zones [3]. An estimated 70,000 to 110,000 people die from malaria each year, the majority being children under five years of age [4].

Like elsewhere in Sub Saharan Africa, the main malaria control interventions in Uganda are chemotherapy and vector control using insecticide-treated bed nets and indoor residual spraying using recommended insecticides [3,5]. Household use of Insecticide-treated bed nets (ITNs) has been widely adopted as a potential solution to the malaria problem in Africa and other malaria endemic regions of the world because of their effectiveness as a physical barrier to break vector-human contact and in reducing vector populations in communities [3-6]. The main entomological justification for the use of ITNs was that most biting by the anthropophilic, endophagic and endophilic vector mosquitoes occurred at hours of the night when most people were in bed and under nets if they had them [7]. Peak-biting activity of *Plasmodium*-transmitting mosquitoes in Uganda is known to be between 22.00 and 05.00 hours, and the majority of mosquitoes bite humans indoors and rest indoors [8]. Despite extensive coverage and prolonged use of ITNs in Uganda, malaria-related morbidity and mortality remains high. Reasons for this are thought to include change in the biting pattern of a greater proportion of the malaria vectors to biting earlier or later in the night and biting outdoors when many people are not in bed, rendering bed nets less effective [6,7], hence causing an increase in the malaria infection rates. If so, this may perhaps explain the continued high rates of morbidity and mortality due to malaria in Kamuli district, and possibly other parts of the country.

To-date only a few studies have been undertaken to examine a comprehensive package of behavioral aspects in areas where ITNs have been used for many years. Other than the development of resistance against the insecticides used that has been reported in several parts of Africa [9,10], Brooke Pers. Communication, 2006), other effects may include changes in biting behaviour expressed by outdoor biting and/or time of biting [11] as well as change in host preference as the favoured host is under the ITN [12].

We set out to investigate the biting activity of *Anopheles* g*ambiae* and *An. funestus* mosquitoes in Kamuli district including an area that had used ITNs consistently for more than five years. Knowledge of the biting patterns and seasonal abundances of the vectors could give guidance on suitable times, locations, and periods of application of other mosquito control interventions such as insecticidal (aerosol) spraying, residual insecticide spraying to supplement insecticide-treated bed nets in the context of integrated vector management. This could also delay the appearance of behavioural changes as have occurred following indoor residual spraying with organochlorines [12,13] in many parts of the world.

The results may contribute valuable data for formulating malaria control policies and plans, particularly in the event of the global efforts (and by Uganda government) to scale up ITN use in the control of vectors of malaria parasites. The results will also provide baseline entomological data upon which impact of prolonged use of ITNs in Kamuli district may be assessed.

## Methods

### Study area

The study was conducted in Kamuli district, one of the malaria endemic districts in Eastern Uganda, located 68 km North of the source of River Nile. The study area was divided into intervention zone (five villages using ITNs for at least five years) and non-intervention.

Zone (five villages not using ITNs). The intervention villages were located in Kamuli Town Council and Nabwigulu Sub County, both in Bugabula County. The ITNs coverage in 5 out of 18 sub counties of Kamuli district by the year 2006 was as follows: Kamuli Town Council (3051 nets in 4080 households), Nabwigulu (3378 nets in 297 households sampled), Balawoli (1066 nets in 256 households sampled), Kitayunjwa (1485 nets in 136 households sampled), and Namwendwa (846 nets: 8264 people in 229 households sampled). The proportion of households that were using bed nets for the last five years in the two sub counties studied (Kamuli Town Council and Nabwigulu) was at least 52%, while at the time of the study coverage stood at 74.8% and 64% for Kamuli Town Council and Nabwigulu respectively, with an average of 69% of the households using at least one net (Kamuli District Health Status Reports, 1999/2000-2004/2005; Kamuli District Health Sector Strategic Plan 2005/06-2010; Personal survey of existing Kamuli Town Council HMIS Reports, 2004/2005-2006; Kamuli District HMIS Reports, 2005/06-2010; Kamuli Christian Children’s Fund, CCF, office records, 2004/2005-2006- all Unpublished).

These villages were privileged with a number of Non Governmental Organizations such as The Christian Child Fund and Plan-Uganda that have intervened with insecticide-treated bed nets since the late 1990s and later with the supply of Long Lasting Nets to supplement the Ministry of Health efforts in the control of malaria targeting pregnant mothers, children under five years and People Living with HIV/AIDS. The Non Governmental Organizations also carried out several community sensitizations in conjunction with the District Health department aimed at promoting ITN use. This is why Kamuli district was chosen for the study.

The non-intervention villages were located in Bugaya and Buyende sub counties, both in Budiope County, in the North East of Kamuli Town Council, and well over twenty kilometers away, with households owning no bed nets before the entomological survey (Kamuli District Health Status Reports, 1999/2000-2004/2005; Kamuli District Health Sector Strategic Plan, 2005/06-2009/10-Un published; Personal House hold Survey).

Both Bugabula and Budiope counties were located in the same climatic zone [14] and were surrounded by a variety of vegetation types including swamps, crop fields and grazing lands. Kamuli district has two rainy seasons, the heaviest rains in March to June and light rains in.

August to November, with a dry spell from December to March (Annual average rainfall: 750 mm to 1500 mm; average maximum temperature: 27°C to 30°C; average minimum temperature 10°C to 20°C and relative humidity: 70 to 80%;) [Source: Kamuli District Agriculture Reports, 2005/06-2009/10-Unpublished]. High mosquito densities and malaria transmission occurred throughout the year (Kamuli District Health Sector Strategic Plan 2005/06-2010-Unpublished).

There was no baseline entomological data at the time of the study since no prior study had recently been conducted in the area. Therefore, this study will provide baseline entomological data for future entomological monitoring surveys to assess the impact of ITNs on the population dynamics of the malaria vector species in Kamuli district.

### Mosquito collections

From January to December 2010 hourly indoor biting mosquitoes were collected from 19.00 to 07.00 hours for four consecutive nights per month by a two-person team of trained catchers using bed net traps [1]. The bed net trap was made by making a 3 × 3 inch hole on each of the sides of an untreated bed net, making a total of 4 to 6 holes on the net. The catcher sat under the bed net trap which gave him some protection which is denied when the human-landing catch method is used. This method was preferred to the CDC light trap because most of the mosquitoes caught in the light trap were found dead and brittle, making morphological identification of the samples difficult. Outdoor human biting catches were carried out concurrently using the same method at the same household ten metres away [1]. People living in a room were protected with an untreated net each, and as hungry mosquitoes persisted in their attempts to look for a blood meal, they got near to the human-baited trap and were caught [15] by the human bait (collector) using an aspirator and a torch [1]. It was assumed that the mosquitoes that entered a trap during any hour were those actively seeking hosts, and, in most cases, would bite human hosts in the same hour and room/house if the bed net trap was absent [7]. The indoor and outdoor human-biting fraction of the *Anopheles* mosquitoes (and time of biting) were determined and recorded throughout the whole sampling period for both intervention and non-intervention zones.

Each hourly catch was placed separately in a disposable polystyrene container pre-labeled with date, time and location of capture and taken to the laboratory for identification of mosquitoes collected [16]. Mosquitoes were kept alive by providing them with a 10% sugar solution to feed on administered through a cotton wick [17]. Each hourly catch of the human-biting fractions of the mosquito population was identified morphologically using a simplified key adopted from Gillies and Coetzee [18], while the morphological identifications were confirmed by an Entomologist at the Vector Control Division, Ministry of Health, Uganda.

The indoor resting blood-fed anophelines were squashed on DNA- binding filter paper, dried and kept at – 20°C for later blood meal analysis [11,16].

### Human biting densities and rates

The human biting densities and biting rates were calculated separately for indoor and outdoor catches. Indoor and outdoor mosquito biting densities were calculated as the sum of the female anopheline catches caught in the villages during the 12-month sampling period divided by the total number of houses sampled for night-biting mosquitoes in the villages. The indoor and outdoor human biting rates were calculated as the total number of mosquitoes caught biting humans during the 12-month sampling period divided by the number of people bitten (the catchers) divided by the number of nights of catching (number of mosquitoes per human per night) [1].

### Meteorological data

Rainfall totals were collected and recorded at a meteorological station in the study area on a monthly basis as daily average temperatures and relative humidity were also recorded during the sampling nights.

### Statistical analysis

Comparison between indoor and outdoor human biting times of the *Anopheles* species in Bugabula and Budiope Counties was carried out using Multi-way analysis of variance (MANOVA). The numbers of mosquitoes by species caught biting humans during the different hours of the night, the indoor and outdoor human biting densities and biting rates of the *Anopheles gambiae* complex and *An. funestus* group of mosquitoes for the whole sampling period were compared within the two counties using the Kruskal-Wallis rank sum test of the R-Statistics soft ware, version 2.15.0 (2012.03.30) [19]. Correlation between the *Anopheles* species (*An. gambiae s.l*. and *An. funestus* group) abundances and monthly rainfall totals in both Bugabula and Budiope counties was established using Pearson correlation of the Minitab Version 15 [20].

### Ethical issues

Prior to start of the study, approval was obtained from the Uganda National Council for Science and Technology and Health Research Ethics Committee (Reference Number: HS 263). House hold owners, village and district authorities were sensitized prior to the study and their permission obtained, while the privacy and psycho-social needs of the individual participants and household members were highly protected. Catchers were selected from the local community to facilitate acceptance from residents. Informed consent was obtained from each catcher. The catchers were trained to collect landing mosquitoes prior to blood feeding to minimize the risk of malaria transmission. They were given anti-malarial drugs as this geographical area has high transmission of *Plasmodium falciparum* with resistance to anti-malarial drugs [Dr. Lopita Micah, Pers. Communication]. At least two bed nets (LLINs) were donated to each participating household following the study.

## Results

Human biting and indoor resting catches of *An. gambiae s.l*. and *An. funestus* mosquitoes A total of 3,519 female *Anopheles* mosquitoes were collected in the 48 households in 1536 man nights. Over 70% of the *Anopheles* mosquitoes caught were *Anopheles gambiae s.l*. as shown in Table 1 below.

**Table 1:** **Human biting and indoor resting catches of female *Anopheles *** **mosquitoes in both Budiope and Bugabula Counties over a 12 month sampling period**

**Mosquito group**	**Budiope County**		**Bugabula County**			
	**Indoor**	**Outdoor**	**Indoor**	**Outdoor**	**Totals**	**%**
*Anopheles gambiae s.l.*	853	1079	299	346	2,577	73.2
*Anopheles funestus*	453	411	39	39	942	26.8
**Totals**	**1**,**306**	**1**,**490**	**338**	**385**	**3**,**519**	
	(114)		(73)			

Number of indoor resting blood-fed anophelines in both countries in parentheses.

There were approximately four times more *Anopheles* mosquitoes caught biting humans in Budiope (Table 1) than in Bugabula county (Chi-squared = 159.894, df = 1, p < 0.001). For both counties, *An. gambiae* catches exceeded those of *An. funestus* (Chi-squared = 86.662, df = 1, p < 0.001), this trend being greater in Bugabula county.

### Biting patterns of the anopheles mosquitoes

**Figure 1:**
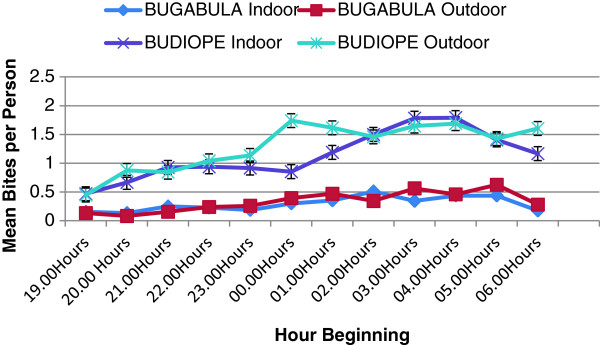
**Indoor and outdoor mean bites of female *Anopheles *** **mosquitoes per person at different hours of the night in Kamuli District.**

**Figure 2:**
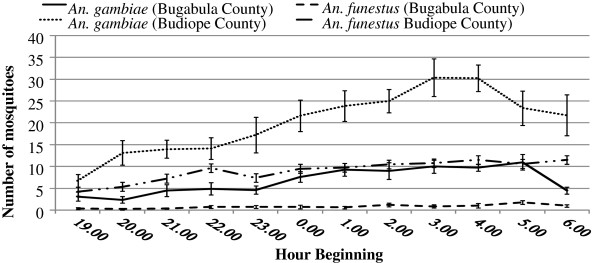
**Hourly catches of female *Anopheles gambiae s.l***. **and *An. funestus *group at different hours of the night in Kamuli District.**

The majority of anopheline bites occurred in the last third of the night (03–00 to 06.00 hours) in both Bugabula and Budiope counties, showing no significant difference in the indoor and outdoor human biting times of the *Anopheles* species in the two counties (Sum Sq = 135.9, df = 11, F = 0.4016, pr =0.954762) (Figures 1 and 2). Peak night biting was observed between 23.00 and 05.00 hours for both *Anopheles gambiae sensu lato* and *funestus* group.

**Figure 3:**
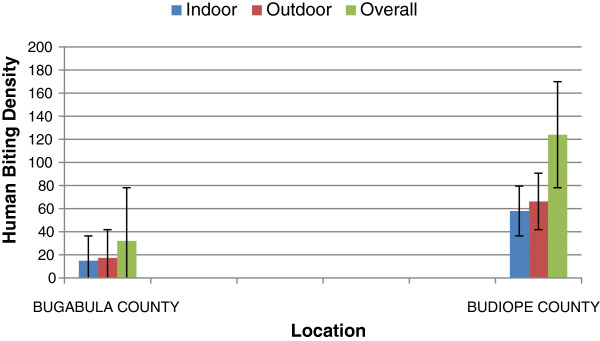
**Indoor and outdoor Human Biting Densities of *Anopheles *** **species in Bugabula and Budiope counties.**

Outdoor biting densities of *Anopheles* species exceeded the indoor biting densities throughout the night in both Bugabula and Budiope counties (Sum sq = 127.2, df = 1, F = 4.1347, p < 0.05) (Figure 3), although the indoor and outdoor human biting densities of *An. funestus* group were apparently equal in both zones (Table 1).

**Figure 4:**
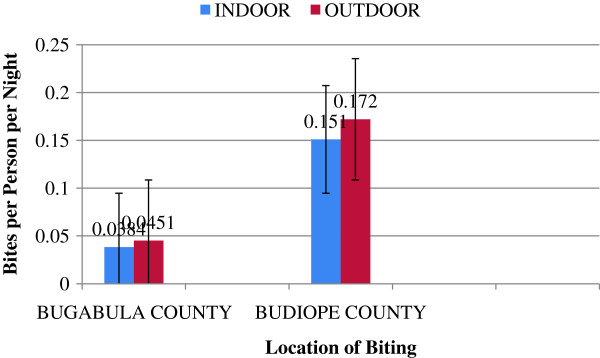
**Human biting rates of *Anopheles *** **species in Bugabula and Budiope Counties, Kamuli district.**

The outdoor biting rates of the anophelines appeared to exceed the indoor biting rates in both zones, however, statistically there was no significant difference shown between the outdoor and indoor human biting rates (Chi-squared = 0.227, df = 1, p =0.634), (Figure 4).

### Seasonal abundances of anopheles gambiae s.l. and anopheles funestus mosquitoes

**Figure 5:**
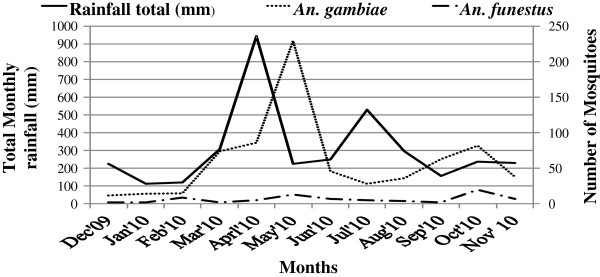
**Monthly abundances of Human**-**biting *Anopheles species *in Bugabula County, Kamuli District, Uganda.**

**Figure 6:**
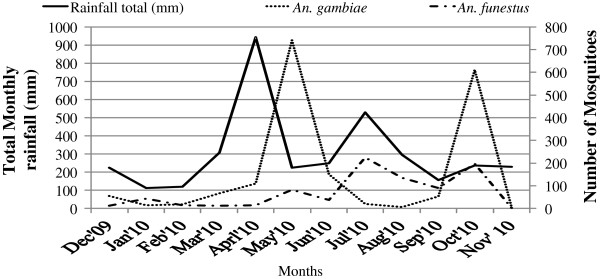
**Monthly abundances of human**-**biting *anopheles species *in Budiope County, Kamuli District, Uganda.**

Human biting catches of *Anopheles gambiae s.l*. peaked during May 2010, with a lower peak occurring in October. Both peaks of the *Anopheles gambiae s.l*. followed the peaks of the major and minor rainfall seasons, (February to May) and (June to September) respectively. The *Anopheles funestus* group of mosquitoes was more abundant during October 2010 in the Bugabula county at the end of both the major and minor rainfall seasons, (Figure 5), while in the Budiope county; this group of mosquitoes was more abundant during July and October 2010 at the end of the minor rainfall season of the year (Figure 6).

Generally, *An. funestus* were prevalent all the year round and were more abundant in the villages of Budiope county than in Bugabula villages. In Bugabula county, there was a correlation between catches of *An. gambiae s.l*. and monthly rainfall totals (r = 0.119; p = 0.713), while there was no correlation between the *An. funestus* catches and monthly rainfall totals (r = -0.099; p = 0.761). This implied that while the abundance of *An. gambiae s.l*. was dependent on the amount of rainfall, with increases in catches corresponding to the increase in rainfall, the *An. funestu*s group could thrive with or without rain fall. In Budiope county, there was no correlation between catches of *An. gambiae s.l*. and monthly rainfall totals (r = -0.077; p = 0.811), while there was a weak correlation between the *An. funestus* catches and monthly rainfall totals (r = 0.066; p = 0.839). This implied that generally, both *An. gambiae s.l*. and *An. funestus* mosquitoes in this zone could thrive all year round regardless of the amount of rainfall.

The monthly average temperatures and relative humidity in the study area were 26.2°C and 64.7% respectively, which were within the range of tropical conditions and the study area in particular.

## Discussion

The results showed low mosquito abundance in Bugabula County, which was probably suggestive of effectiveness of the vector control intervention (ITNs/ LLINs) under use in this zone. However, this could not rule out the fact that there could be other prevailing factors in the area other than the ITNs.

The results in Bugabula County may be comparable to results of a study on impact of >5 years of 60-86% coverage with insecticide treated bed nets on malaria transmission indices on the south coast of Kenya in which the density, among other transmission indices, was reduced for both *An. gambiae* and *An. funestus* mosquitoes [21]. In Western Nyanza province, Kenya, decline in the population of *Anopheles gambiae* mosquitoes was also associated with increase in household ownership of insecticide-treated bed nets [22].

There was a relatively higher proportion of the *An. funestus* group in Budiope county. This could be attributed to the presence of more permanent water for breeding provided by a larger Nabigaga swamp in this locality. *Anopheles funestus* is known to breed all year round and prefer permanent, stagnant water bodies such as shores of rivers and creeks, swamps or fish ponds for breeding, while *An. gambiae* complex breed in temporary/man-made water bodies e.g. pools, puddles or brick pits, fields, construction sites, hoof prints or even tyre tracks [8].

A study on malaria-infective biting hours of the night estimated that as many people in tropical areas get up before dawn, protection by bed nets would be most reliable against mosquitoes biting between 23.00 and 05.00 hours, with many people exposed before and after those hours [7]. The occurrence of the majority of biting *in* the last third of the night (during 03.00 to 06.00 hours) and peak biting by *Anopheles gambiae* and *An. funestus* between 23.00 and 05.00 hours is consistent with the already known time of peak biting by the majority of human-biting sporozoite positive *Anopheles gambiae* and *An. funestus* mosquitoes, i.e. between 23.00 and 05.00 hours, a period when most people are in bed and under nets if they have them [7,23].

The fact that most of the bites from *An. gambiae sensu lato* and *An. funestus* occur during hours of the night when most people are in bed was the source of the enthusiasm for the use of insecticide-treated bed nets for malaria control in Africa [7]. This, combined with the lack of a significant difference in the time at which human biting by the *Anopheles* species in both Bugabula and Budiope Counties occurred (p = 0.9547), indicated that insecticide-treated materials i.e. Long Lasting insecticide-treated nets, curtains, etc. could be an effective control method in the study area.

The present study shows that ITNs have not had an effect on the biting times and seasonality of the *Plasmodium*-transmitting mosquitoes other than the mass killing effect shown in Bugabula County. These results are comparable to the study in Western Kenya which demonstrated that wide use of bed nets in the short term at least reduced human vector contact and blood feeding success but did not lead to changes in the biting times of the *plasmodia* vectors [24].

The results of the present study, however, contrast with results of some other studies, for example, in another study in Kenya, biting occurred earlier in the evening following ITN use [11]. In Papua New Guinea and Tanzania studies, shifts in time of biting were observed where mosquito biting occurred earlier in the evening as hosts had not yet gone to bed and were easily accessible [12]. In a study to test bed net traps for monitoring mosquito populations and time of biting in Tanzania and possible impact of prolonged use of insecticide treated bet nets, it was observed that somewhat more of the *Anopheles* biting occurring early and late in villages with ITNs, whereas in villages with no history of ITN use, biting was concentrated in the middle of the night. This suggested that behavioural adaptation to avoid contact with ITNs could have started to evolve in those ITN villages [25]. Therefore, the contrasting results in the present study could probably further indicate that ITNS are still protective, as peak biting by the vector mosquitoes still occurred at hours of the night when people are believed to be in bed.

The study also reveals that ITN use has not had an effect on the endophilic behaviour of the *Anopheles gambiae* and *An. funestus* mosquitoes [26] as evidenced by the proportionately equal indoor resting blood-fed mosquito catches in both zones, Bugabula county (n = 73) and Budiope county (n = 114) in Table 1. The endophagic and endophilic behaviours of the vectors of *Plasmodium* parasites have been the basis of deploying ITNs and IRS in controlling malaria in Africa [27].

In Papua New Guinea and in Kenya, as well as in Tanzania, a shift to outdoor biting was observed following wide ITN use [12]. Malaria vector feeding and resting behaviours are likely to have changed to maximize available feeding opportunities [27]. In the present study, outdoor biting densities of *An. gambiae* exceeded indoor biting densities throughout the night in both zones (p <0.05), while indoor biting generally exceeded outdoor biting catches for the *An. funestus group*. This trend, particularly for the *gambiae* complex apparently could not be a result of prolonged use of ITNs. This pattern could be due to possible physiological other than behavioural resistance in the *Anopheles gambiae* mosquito population in the area, arising from other factors other than ITN use [12].

There was no significant difference between outdoor and indoor biting rates (Bites per person per night) of the *Anopheles* species in both zones (p = 0.634). This trend implied that probably at one time, the outdoor human biting rates equaled the indoor biting rates.

Higher catches of the *Anophele*s species were realized during the rainy seasons (March to May and July to October) or at onset of the rains (Figures 5 and 6), this being attributed to the fact that the rainy season provides water for breeding [28] and increases humidity for mosquito survival [13]. Densities of *Anopheles gambiae s.l*. were observed to increase following peak rainfall (April and July 2010) in both zones, while *An. funestus* densities increased at the end of the rainy season and at the beginning of the dry season. This finding could explain the all-year-round malaria transmission as the two groups of mosquitoes peak at different times and hence prolonging the transmission period through the year in Kamuli district and most parts of Uganda.

There were more mosquitoes of the An. *funestus* group caught in the villages of Budiope County near Nabigaga swamp than in Bugabula villages which were also surrounded by a seasonal swamp located between Kamuli Town Council and Nabwigulu Sub County. Nabigaga swamp provides more permanent mosquito breeding grounds throughout the year, preferred by *An*.*funestus* mosquitoes [23,26]. Therefore, even during the dry periods of the year this swamp is continuously productive for *An. funestus* mosquitoes. This explains the insignificant correlation between *Anopheles* catches, particularly of the *An. funestus* group of mosquitoes, and rainfall in both zones, although other factors such as the presence of small scale irrigation agriculture, aquaculture (in Bugabula County) and bore hole grounds with water collection points that could provide *Anopheles* breeding grounds could be important attributes. Humans are therefore exposed to mosquito bites, which are probably malaria sporozoite-positive, all year round as the *Anopheles gambiae* and *An. funestus* species that are highly anthropophilic and endophilic [13,26,28] are prevalent throughout the year particularly in Budiope County.

Results of the study show that people who wake up before dawn particularly those who wake up early for trade and farming activities in the urban/semi-urban centres and rural areas respectively are at risk of getting many mosquito bites as they get exposed to the later biting cycle of the vectors, with higher chances of contracting the infections they transmit, namely: *Plasmodia* and filariases [26]. It is also probable that the observed mosquito biting patterns extend to as far as the shores of lake Kyoga and areas along the River Nile, implying that the fishing communities therein are exposed to a lot of mosquito bites as they engage themselves in various all-night fishing activities. Further study is required in the areas along the River Nile and shores of lake Kyoga to establish the human-biting patterns of *Anopheles* mosquitoes and the epidemiology of malaria in these areas.

## Conclusion

The findings in the present study provide useful information on the biting patterns and seasonal abundances of *Anopheles gambiae* and *An. funestus* mosquito species in Kamuli district, Uganda. This may be a basis for formulating appropriate malaria control interventions in the area. The use of Long Lasting insecticide treated bed nets as a control strategy for malaria in Kamuli district and Uganda as a whole could be still an effective intervention as the *Anopheles* host-seeking activity peaked between 23:00 and 05:00 hours and the majority of biting occurred between 03:00 and 06:00 hours when most people are in bed.

Since *An. gambiae* sensu *lato* and *An.funestus* group identified in the study area are known for their highly anthropophilic, endophagic and endophilic behaviour (feeding on humans indoors and resting indoors) [26,28], the use of insecticide treated Long Lasting nets (LLINs) and indoor residual spraying (IRS) using recommended cost-effective and ecologically friendly insecticides may be the appropriate vector control strategies for this study area. The Roll Back Malaria target of 80% [9] or even universal coverage [2] of LLINs should be aimed at by Uganda’s National.

Malaria Control Programme coupled with mass sensitization on bed net ownership, their proper and consistent use so as to benefit from the mass killing effect obtained from massive use of LLINs. However, if LLINs are to be massively used against malaria in Uganda and the entire Sub-Saharan Africa region, their effects on mosquito biting patterns (and insecticide susceptibility) need to be regularly monitored [6].

Mosquito control by indoor residual spraying should be implemented around the months of January/February and June/July before the onset of the rainy seasons, that is, ahead of the peak human biting densities.

In addition to IRS and LLINs, larval source management strategy (which includes larviciding and source reduction) presents another potential intervention that may be promoted in this part of the country in the context of integrated vector management strategy [23,26,29,30]. The immune suppressed groups of people (pregnant women, children under five years of age and the HIV/AIDS patients) should be particularly taken care of. House improvements to reduce indoor human biting densities should also be encouraged [26,31].

## Competing interests

The authors declare that they have no competing interests.

## Authors’ contributions

FGK conceived, designed and carried out the mosquito survey, analysed the data and drafted the manuscript. AMA and JBK helped to design the study and provided backstopping during the field work and provided critical comments on the manuscript. AO helped to design the study and provided critical comments on the different versions of the manuscript. All the authors read and approved the final manuscript.
